# Plant Diversity and Fungal Richness Regulate the Changes in Soil Multifunctionality in a Semi-Arid Grassland

**DOI:** 10.3390/biology11060870

**Published:** 2022-06-06

**Authors:** Zhuo Li, Xiaowei Liu, Minghui Zhang, Fu Xing

**Affiliations:** 1 Key Laboratory of Vegetation Ecology, Institute of Grassland Science, Northeast Normal University, Ministry of Education, Changchun 130024, China; liz347@nenu.edu.cn (Z.L.); liuxw101@nenu.edu.cn (X.L.); zhangmh740@nenu.edu.cn (M.Z.); 2 Jilin Songnen Grassland Ecosystem National Observation and Research Station, Changchun 130024, China

**Keywords:** plant diversity loss, soil multifunctionality, fungal diversity, saprotrophic fungi, rare microbial taxa

## Abstract

**Simple Summary:**

Understanding relationships between biodiversity and ecosystem functions is important in the context of global plant diversity loss. We evaluated the relationships between soil bacterial and fungal diversity, rare microbial taxa, and soil multifunctionality in a semi-arid grassland with varied plant diversity levels. The fungal richness rather than bacterial richness was positively related to soil multifunctionality. The relative abundance of saprotrophs was positively correlated with soil multifunctionality, and the relative abundance of pathogens was negatively correlated with soil multifunctionality. Furthermore, the rare fungal taxa played a disproportionate role in regulating soil multifunctionality. The shift of plant biomass allocation patterns increased plant below-ground biomass in the high diversity plant assemblages, which can alleviate soil microbial carbon limitations and enhance the fungal richness, thus promoting soil multifunctionality. This study provides a new perspective for evaluating the relative roles of fungal and bacterial diversity in maintaining multiple soil functions under global plant diversity loss scenarios.

**Abstract:**

Loss in plant diversity is expected to impact biodiversity and ecosystem functioning (BEF) in terrestrial ecosystems. Soil microbes play essential roles in regulating ecosystem functions. However, the important roles and differences in bacterial and fungal diversity and rare microbial taxa in driving soil multifunctionality based on plant diversity remain poorly understood in grassland ecosystems. Here, we carried out an experiment in six study sites with varied plant diversity levels to evaluate the relationships between soil bacterial and fungal diversity, rare taxa, and soil multifunctionality in a semi-arid grassland. We used Illumina HiSeq sequencing to determine soil bacterial and fungal diversity and evaluated soil functions associated with the nutrient cycle. We found that high diversity plant assemblages had a higher ratio of below-ground biomass to above-ground biomass, soil multifunctionality, and lower microbial carbon limitation than those with low diversity. Moreover, the fungal richness was negatively and significantly associated with microbial carbon limitations. The fungal richness was positively related to soil multifunctionality, but the bacterial richness was not. We also found that the relative abundance of saprotrophs was positively correlated with soil multifunctionality, and the relative abundance of pathogens was negatively correlated with soil multifunctionality. In addition, the rare fungal taxa played a disproportionate role in regulating soil multifunctionality. Structural equation modeling showed that the shift of plant biomass allocation patterns increased plant below-ground biomass in the highly diverse plant plots, which can alleviate soil microbial carbon limitations and enhance the fungal richness, thus promoting soil multifunctionality. Overall, these findings expand our comprehensive understanding of the critical role of soil fungal diversity and rare taxa in regulating soil multifunctionality under global plant diversity loss scenarios.

## 1. Introduction

Soil supports a wide range of ecological functions and services that are important to human welfare, and soil multifunctionality can be used as a comprehensive index to evaluate soil quality [1,2,3,4]. Multifunctionality is an essential biological and management concept that describes the ability of an ecosystem to maintain multiple ecological functions simultaneously [1,2,5]. As significant drivers of ecosystem functions, soil microbial communities can be regulated by plant diversity via changing the nutrient availability and microenvironmental conditions [6,7,8]. However, global climate changes and intensive anthropogenic activities have led to the loss of plant diversity in grassland ecosystems [2,9,10,11]. Therefore, there remains an urgent need to understand the mechanisms by which soil microorganisms drive soil multifunctionality in the context of global plant diversity loss.

Below-ground microbes comprise a large portion of global terrestrial ecosystem life’s genetic diversity and support important ecosystem functions and services, such as biomass production, nutrient cycling, and decomposition of organic matter [10,12,13]. Additionality, soil microorganisms are an essential link between the above-ground and below-ground components of terrestrial ecosystems [14,15,16,17]. Bacteria and fungi constitute vital parts of the soil microbiome [18]. Nevertheless, bacteria and fungi play different roles in regulating ecosystem processes [14]. In detail, bacteria are the essential drivers of soil N cycle processes, such as N_2_ fixation, nitrification, and denitrification [19,20,21]. However, other studies have suggested that soil fungal communities might dominate in driving soil functions [14]. Soil fungi can decompose recalcitrant plant litter efficiently and form links with roots to capture and transport subsurface carbon (C) [22]. For instance, saprophytic fungi are primary mediators for the decomposition of plant litter, and their mycelium networks across the soil litter interface and networks are a highly dynamic channel through which nutrients can be easily distributed [23]. Despite the above studies, most research has concentrated on how soil microbes affect individual or specific nutrient cycling functions. However, few studies have evaluated the relative significance of bacterial and fungal diversity in regulating soil multifunctionality in the context of plant diversity in a semi-arid grassland.

Furthermore, the effects of rare microbial taxa on soil functions have often been ignored in most previous soil multiple functions studies because they have mainly concentrated on predominant taxa; a majority of rare microbial taxa were frequently eliminated from original datasets. However, rare microbial taxa have important ecological roles because these may be activated to perform critical functions under environmental changes [24,25]. For instance, rare taxa were found to play essential roles in nutrient cycling after disturbances in aquatic ecosystems [26]. Rare microbial taxa have also been used as indicators of changes in ecosystem functions under long-term greenhouse cultivation conditions in subtropical agricultural soils [27]. Therefore, rare microbial taxa can represent a microbial “seed bank” that may be activated when the environment is disturbed [24,28]. The intensity and frequency of plant diversity loss due to global climate change are expected to increase in the future [9,29,30]; therefore, it is of great significance to link the ecological functions of rare microbial taxa with soil multifunctionality. Unfortunately, relevant knowledge is still scarce, especially in semi-arid grassland ecosystems. Such knowledge is critical for developing a management framework to maintain rare taxa involved in functionality and decrease the impact of future climate change on a semi-arid grassland.

Loss in plant diversity of terrestrial ecosystems might aggravate C and other nutrient limitations [30,31,32]. Heterotrophic organisms depend on plants to obtain C substrates; therefore, there is ample evidence that soil microbes are usually limited by C [33]. Microorganisms play an essential role in ecosystem functions [6,18,34]; thus, more research is undoubtedly required to evaluate how the shift in microbial nutrient limitations affects microbes under changes in plant diversity. Soil enzymes produced by microbes transform substrates in soil C and nutrient cycles [35]. Thus, enzymatic stoichiometry provides a method for this study [35]. Several studies have shown that less diverse plant communities allocate fewer photosynthates to below-ground ecosystems by decreasing the exudation of root exudates [32]. Root exudates are an important C source for microbial growth [36]; therefore, a less diverse plant community might intensify microbial C limitation [37]. C limitation could affect microbial biosynthesis processes [35]; thus, lower below-ground biomass allocation would reduce soil functions such as microbial growth and respiration by increasing microbial C limitation [36,38]. The above results show that plant diversity affects soil microbial communities by trophic interactions and altering soil biochemical processes [12,13]. Thus, the loss of plant diversity may alter the relationships between plant and soil microbial communities. Considering that soil microbial communities mediate ecosystem functions, it is essential to elucidate the mechanisms by which plant-diversity-induced changes in microbial C limitation regulate soil multifunctionality through impacting soil microorganisms in a meadow grassland.

In addition, ecosystem multifunctionality is different from ecosystem services multifunctionality in that the former represents the overall performance of an ecosystem without considering stakeholders; whereas the latter is defined as the ability of ecosystems to synergistically provide various ecosystem functions and services that translate into a variety of social benefits and welfare [1]. Therefore, investigations of ecosystem service multifunctionality have significant importance in future semi-arid grassland ecosystems management and sustainable development. In this study, a meadow grassland in northeast China was selected to discuss the soil multifunctionality and microbial driving mechanisms under a plant diversity loss scenario. The objectives of this study were to 1) evaluate the important roles of soil bacterial and fungal diversity and rare taxa in driving soil multifunctionality under different plant diversity conditions; 2) reveal the underlying mechanisms by which plant-diversity-induced alterations of microbial C limitation regulate soil multifunctionality through soil microorganisms; 3) provides a scientific reference for the evaluation of semi-arid grassland ecosystem services multifunctionality.

## 2. Materials and Methods

### 2.1. Study Site

**Figure 1:**
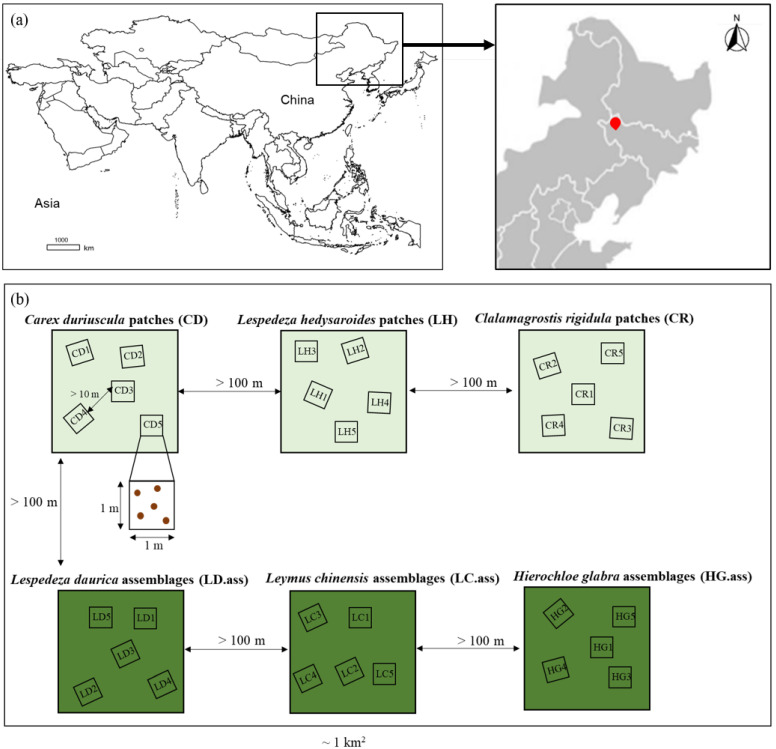
Locations of the study site (**a**) and experimental design for plant and soil sampling (**b**) in the Songnen grassland, China. Three single plant species patches, i.e., *Carex duriuscula* C.A.Mey. (CD), *Lespedeza hedysaroides* (Pall.) Kitag. (LH), and *Calamagrostis rigidula* A.I.Baranov and Skvortsov (CR) assemblages and three multiple species coexisting plant assemblages with different dominant *Lespedeza daurica* (Laxm.) Schindl. (LD.ass), *Leymus chinensis* (Trin.) Tzvelev (LC.ass), and *Hierochloe glabra* Trin. (HG.ass) naturally existing in the grassland. For the six study sites selected above, the interval between every two sites was greater than 100 m. For plant and soil sampling, five plots (1 m × 1 m) with an interval of more than 10 m were randomly selected at each study site. Five soil cores were randomly selected in each plot by a soil auger.

The experiment was conducted at the Tongyu Observatory in a semi-arid climate and environment (44° 25′ N, 122° 52′ E). This site is located within the Songnen grassland ecosystem of northeastern China (Figure 1a). The vegetation is a meadow steppe, situated at the eastern end of the Eurasian grassland belt with *Leymus chinensis* (Trin.) Tzvelev (https://www.ipni.org/) (accessed on 1 June 2022) is the dominant species. The soil texture is clay loam, according to the International Society of Soil Science Standard. The study area has a temperate continental monsoon climate. The mean annual precipitation is ~404 mm, and more than 80% of the rainfall is concentrated during the growing season (from May to September). The mean annual temperature is approximately 5.7 °C.

### 2.2. Experimental Design and Sampling

Within approximately 1 km^2^ of the study area, we randomly selected three single plant species patches i.e., *Carex duriuscula* C.A.Mey., *Lespedeza hedysaroides* (Pall.) Kitag., and *Calamagrostis rigidula* A.I.Baranov and Skvortsov assemblages and three multiple species coexisting plant assemblages with different dominant *Lespedeza daurica* (Laxm.) Schindl., *L. chinensis*, and *Hierochloe glabra* Trin. naturally existing in the grassland. Single species assemblages and multiple species assemblages represent low and high diversity levels, respectively. The plant species composition is shown in Table S1. A vegetation survey was conducted in mid-August 2018 when the standing biomass reached its maximum. For the six study sites selected above, the interval between every two sites was greater than 100 m. For plant and soil sampling, five plots (1 m × 1 m) with an interval of more than 10 m were randomly selected at each study site (Figure 1b). In this study, soil characteristics of low diversity plant assemblages and high diversity plant assemblages were similar (*p* > 0.05) (Figure S1); thus, soils were comparable across different plant diversity plots. We selected six sites with similar soil characteristics but different levels of plant diversity. It is reasonable to obtain data based on one-time sampling to explore the impact of different levels of plant diversity on soil, microorganisms, and soil functions. Additionally, this method has been widely adopted by many studies in the field of ecology [32,39].

All the plant species were identified and recorded in each plot; then, soil and plant samples were collected simultaneously from each plot. Five soil cores (0–10 cm) were randomly selected in each plot by a soil auger (5 cm diameter), then mixed to form a composite sample. Next, the soil samples were sieved (2 mm) to remove any roots and stones. We harvested above-ground living plants to evaluate each plot’s above-ground biomass (AGB). The below-ground biomass (BGB) of each plot was measured by root biomass from three soil cores with a diameter of 10 cm and a depth of 30 cm. All the soil samples were placed in well-sealed zippered bags and transported to the laboratory within 24 h in cooling boxes.

### 2.3. Plant and Soil Samples Analysis

The plant AGB and BGB were determined by plant materials heated at 105 °C for 30 min to rapidly cease metabolic activities [40] and then oven-dried at 65 °C for 48 h until the weight remained constant [41]. The soil samples were divided into three subsamples for physiochemical analyses and the assessment of microbial communities. One aliquot of soil was stored at 4 °C and used to determine the soil water content (SWC), soil available N (AN) content (NH_4_^+^-N and NO_3_^−^-N), soil net nitrification rate (Nn), and the net N mineralization rate (Nm). Another aliquot of soil was stored at −80 °C and used for the analysis of high-throughput sequencing and to assess activities of α-1,4-glucosidase (αG), β-1,4-glucosidase (βG), β-1,4-xylosidase (βX), β-D-cellobiohydrolase (CBH), leucine aminopeptidase (LAP), β-1,4-N-acetylglucosaminidase (NAG), and alkaline phosphatase (ALP). Finally, the third aliquot of soil was air-dried at room temperature for soil pH, electrical conductivity (EC), soil total organic carbon (TOC), total soil nitrogen (TN), total soil phosphorus (TP), and available soil phosphorus (AP) analyses.

The soil pH was detected in deionized water (soil: water = 1:5, w:v) with a portable pH meter (Leichi PHBJ-260, Shanghai, China), and the soil EC was measured with an electronic conductivity meter (Leici DDS307, Shanghai, China) [42]. The SWC was measured as the weight loss recorded after the fresh soils had been oven-dried to a constant weight at 105 °C [10]. The soil TN content was measured with an elemental analyzer (vario EL cube, Elementar, Langenselbold, Germany). Briefly, 30 mg of the air-dried soil was put into a tinfoil cup and tightly wrapped in soil and then put into an automatic sampling tray; the results were analyzed on the machine. Soil TOC was determined with an elemental analyzer (Isoprime 100, Isoprime Ltd., Manchester, UK). The NH_4_^+^-N and NO_3_^−^-N concentrations were measured by extraction with 2 M KCl (soil: water = 1: 5, w:v) and analyzed with a continuous flow analyzer (Futura, Alliance-AMS) [41]. The Nn and Nm were calculated according to alterations in the concentrations of NH_4_^+^-N and NO_3_^−^-N before and after incubation [43]. For soil TP, 0.5 g air-dried soil and ball milling were digested with HClO_4_ (7.7 mL, 75%) at 203 °C for 75 min [44]. Soil AP was extracted with 0.5 M NaHCO_3_, and the molybdenum blue colorimetric method was used to analyze [45]. All enzyme activities were measured using a 4-methylumbelliferyl (MUB) substrate, except for 7-amino-4-methyl-coumarin (7-AMC) for the LAP [46]. Seven enzyme activities were determined in black 96-well microplates. Four replicate wells were set up for each enzyme test sample (200 μL slurry + 50 μL substrate) and corresponding substrate control (200 μL buffer + 50 μL substrate). Four replicate wells were set up for standard fluorescence (200 μL buffer + 50 μL standard), slurry control (200 μL slurry + 50 μL buffer), and quench standards (200 μL slurry + 50 μL standard). The assay plate was incubated in a dark environment at 25 °C for 3 h. Fluorescence was measured using a microplate fluorometer (TECAN infinite F200, Tecan Group, Switzerland) with excitation and emission filters of 360 nm and 460 nm, respectively.

### 2.4. Assessment of Microbial Communities

According to the manufacturer’s protocol, total genomic DNA was extracted from 0.3 g of soil with the PowerSoil DNA Isolation Kit (MO BIO Laboratories, Inc., Carlsbad, CA, USA). The concentration and purity of the extracted DNA were measured with a NanoDrop 2000 spectrophotometer (Thermo Fisher Scientific, Waltham, MA, USA ). DNA quality was evaluated by 1% agarose gel electrophoresis. We acquired information on the diversity and composition of soil bacteria and fungi by performing 16S rRNA and ITS genes amplicon sequencing and the 338F/806R (5′-ACTCCTACGGGAGGCAGCA-3′, 5′-GGACTACHVGGGTWTCTAAT-3′) and ITS1F/ITS2R (5′-CTTGGTCATTTAGAGGAAGTAA-3′, 5′-GCTGCGTTCTTCATCGATGC-3′) primer pairs, respectively. The total volume of the PCR amplification for bacteria and fungi was 50 μL, including 10 μL buffer, 0.2 μL Q5 High-Fidelity DNA Polymerase, 10 μL High GC Enhancer, 1 μL dNTP, a 10 μM concentration of each primer, and 60 ng genome DNA. The thermal cycling conditions were 95 °C denaturation for 5 min, 15 cycles at 95 °C for 1 min, 50 °C for 1 min, 72 °C for 1 min, and 72 °C final extension for 7 min. Ultimately, we quantified all PCR products by Quant-iT™ dsDNA HS Reagent and pooled them together. Furthermore, we conducted a high-throughput sequencing analysis of 16S rRNA and ITS genes by the Illumina Hiseq 2500 platform (2 × 250 paired ends) at Biomarker Technologies Corporation (Beijing, China). Bacterial and fungal sequences were quality-filtered with the QIIME software package and merged using the FLASH software package [47]. The operational taxonomic units (OTUs) were defined by 97% similarity. Representative sequences of bacteria and fungi were annotated with the SILVA and UNITE databases [48]. The final total data set retained 1852 and 1325 OTU numbers and 2,057,790 and 2,071,174 clean reads for bacteria and fungi, respectively. The raw bacterial and fungal reads were deposited into the National Center for Biotechnology Information (NCBI) Sequence Read Archive (SRA) database under accession numbers PRJNA810930 and PRJNA810946, respectively.

### 2.5. Definition of Abundant and Rare Taxa

Previous studies have widely used relative abundance as a metric to describe microbial taxa or OTUs in their environment. Thus, relative abundance is useful for classifying abundant or rare taxa in microbial communities [24,49]. We identified relative abundance thresholds as 1% for abundant taxa and 0.01% for rare taxa [26,27,50]. These classifications ignored intermediate taxa (relative abundance between 0.01 and 1%) and oscillatory taxa (rare and abundant under different environment conditions) [27]. All OTUs were classified into six categories based on the criteria used in recent studies [26,27,50]: always abundant taxa (AAT, relative abundance ≥ 1%); conditionally abundant taxa (CAT, relative abundance ≥ 0.01% in all samples and ≥ 1% in some samples); always rare taxa (ART, relative abundance < 0.01% in all samples); conditionally rare taxa (CRT, relative abundance < 0.01% in some samples but never >1% in any samples); moderate taxa (MT, relative abundance between 0.01% and 1% in all samples); and conditionally rare and abundant taxa (CRAT, relative abundance < 0.01% in some samples and ≥ 1% in some samples). Finally, AAT and CAT were classified as abundant taxa, and ART and CRT were classified as rare taxa [26,27,50].

### 2.6. Assessment of Multifunctionality

Soil multifunctionality was quantified based on ten soil functions widely adopted in previous studies [51,52,53,54,55]. These parameters are related to organic matter decomposition, climate regulation, and nutrient cycling, including αG, βG, βX, CBH, LAP, NAG, Nn, Nm, ALP, and AP. Of them, αG, βG, βX, and CBH represent the C cycle, LAP, NAG, Nn, and Nm represent the N cycle, and ALP and AP represent the P cycle (Table S2). The averaging and threshold approaches have commonly been used to estimate the relationship between biodiversity and multifunctionality [2,34,53,56,57,58]. In this study, we evaluated the soil multifunctionality index using three methods, which contained averaging approach [56], single-threshold, and multiple-threshold approach [57,58]. To obtain an average soil multifunctionality index, each function was standardized by Z-score transformation and then averaged [2,56]. We used a single-threshold approach to calculate the number of soil functions exceeding a given threshold (25%, 50%, 75%, and 90%) [2,54]. However, the multiple-threshold method can demonstrate the effect of bacterial and fungal richness on soil multifunctionality in the full threshold range.

### 2.7. Enzymatic Stoichiometry

We constructed the vector analysis of soil enzymatic stoichiometry to evaluate soil microbial C limitation levels [59]. A longer vector length represents a stronger C limitation.

$$\mathrm{Vector}\;\mathrm{length}\;\left(\mathrm{unitless}\right)=\sqrt{{\left(\ln\beta G/\ln\left(\mathrm{NAG}+\mathrm{LAP}\right)\right)}^{2}+{\left(\ln\beta G/\mathrm{lnALP}\right)}^{2}}$$

### 2.8. Statistical Analysis

We used an independent sample *t*-test to evaluate the differences in plant and soil properties and soil multifunctionality between different levels of plant diversity. When the data did not meet the requirements for normality (Shapiro Wilk test) and homogeneity of variance (Bartlett test), log transformations were performed on the data. We calculated the microbial richness with the “*vegan*” package [60]. Ordinary least squares (OLS) linear regression was conducted to explore the relationships between microbial C limitation and microbial richness; OLS linear regression also was conducted to explore the relationships between bacterial richness, fungal richness, relative pathogen abundance, saprotrophic relative abundance, relative symbiont abundance, and soil multifunctionality [14,15,34,61]. Three methods of soil multifunctionality were evaluated with the “multifunc” package [58]. According to recent studies [14,47,61,62], functions were obtained based on fungal taxa by the FUNGuild database (http://www.stbates.org/guilds/app.php) (accessed on 6 January 2019). Random forest modeling analysis was applied with the “randomForest” package [63] to identify the major statistically significant microbial taxa (OTU level) in impacting soil multifunctionality. Then, seven microbial taxa, including four bacterial taxa and three fungal taxa, were selected in the random forest modeling. The “A3” package [64] was used to assess the importance of each predictor on soil multifunctionality. In addition, we used piecewise structural equation modeling (SEM) with the “piecewiseSEM” [65], “nlme” and “lme4” packages [66] to investigate the potential direct and indirect effects of plant diversity on soil multifunctionality. The prior model was constructed based on current ecological knowledge of the grassland ecosystems [7,36,53,67] to evaluate the link between soil biodiversity and multifunctionality (averaging), assuming that plant diversity altered plant biomass allocation patterns and microbial C limitation, thus promoting soil microbial richness and soil multifunctionality (Figure S2a). Fisher’s C test (0 ≤ Fisher’s C ≤ 2 *df* and 0.05 < *p* ≤ 1.00) was used to verify the rationality of the modeling results. Among significant models, the one with the lowest Akaike information criterion (AIC) value was selected for the final SEM analysis [68] (Figure S2b–d). All statistical analyses and visualizations of this study were performed in R v.4.1.2 software.

## 3. Results

### 3.1. Plant and Soil Properties and Soil Multifunctionality

**Figure 2:**
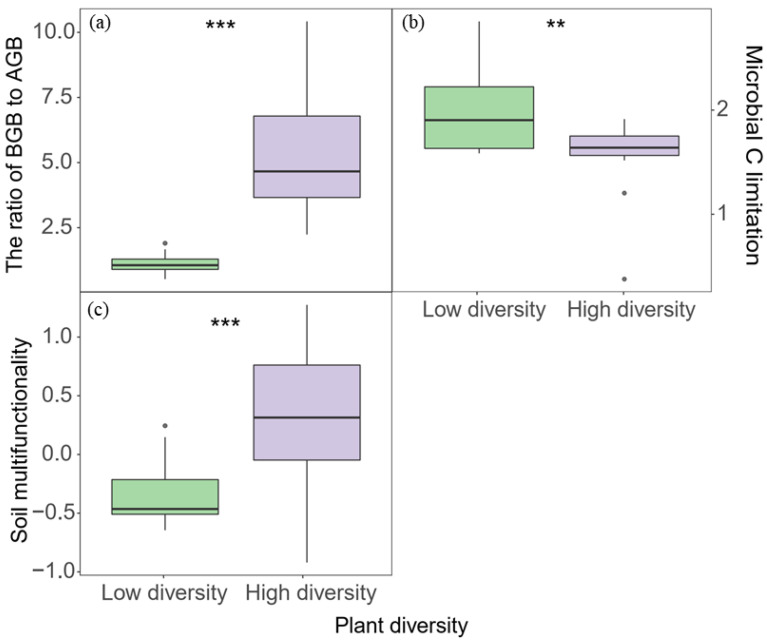
The ratio of BGB to AGB (**a**), microbial C limitation (**b**), and soil multifunctionality (**c**) in response to plant diversity. ** *p* < 0.01 and *** *p* < 0.001 (*t*-test). AGB: above-ground biomass, BGB: below-ground biomass.

The ratio of BGB to AGB was significantly higher with high plant diversity than with low plant diversity (Figure 2a; *p* < 0.001). Microbial C limitation was higher with low diversity than with high diversity (Figure 2b; *p* < 0.01). The results showed that plant diversity significantly altered soil multifunctionality (Figure 2c), i.e., high diversity significantly increased soil multifunctionality compared with low plant diversity (Figure 2c; *p* < 0.001). SWC, soil pH, EC, AN, and TOC showed no significant differences between the two plant diversities (Figure S3a–d,g). Soil TN and TP were higher with low diversity than with high diversity (Figure S3e,f; *p* < 0.05 and *p* < 0.01). In addition, the soil C/N was much higher with high diversity than with low diversity (Figure S3h; *p* < 0.001).

### 3.2. Soil Microbial Community Composition

**Figure 3:**
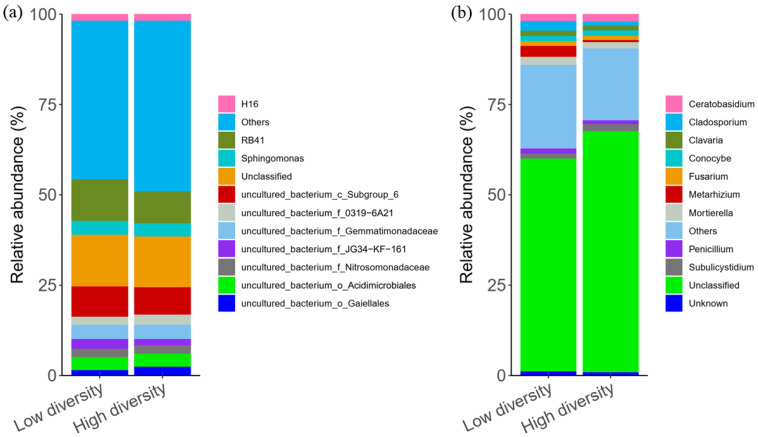
Relative abundance of bacterial (**a**) and fungal (**b**) dominant genera in different plant diversity levels, “Others” represents all genera with relative abundance < 1%.

The rarefaction curves of OTU richness of bacterial and fungal communities almost approached saturation (Figure S4a,b), showing that the amount of data of sequenced reads were reasonable. The bacterial OTUs were assigned to 23 phyla, 76 classes, 110 orders, 188 families, 271 genera, and 226 species; similarly, the fungal OTUs were assigned to 10 phyla, 23 classes, 51 orders, 103 families, 177 genera, and 152 species. The bacterial communities were dominated by Acidobacteria, Proteobacteria, and Actinobacteria (Figure S5). A significantly higher relative abundance of Acidobacteria was observed with low plant diversity than with high plant diversity (Figure S5a; *p* < 0.001). The relative abundance of Actinobacteria and Chloroflexi was higher with high diversity than with low diversity (Figure S5b,d; *p* < 0.001). The fungal communities were dominated by Ascomycota and Basidiomycota (Figure S5). The bacterial communities were dominated by the genera *RB41*, *uncultured_bacterium_c_Subgroup_6*, and *uncultured_bacterium_f_Gemmatimonadaceae* (Figure 3a). However, the fungal communities were dominated by the genera *Ceratobasidium*, *Mortierella*, and *Cladosporium* (Figure 3b). The richness (OTU richness) of fungal and bacterial were significantly greater with high diversity than with low diversity (Figure S6; *p* < 0.001, *p* < 0.05).

### 3.3. Microbial Taxa in Relation to Soil Multifunctionality

**Figure 4:**
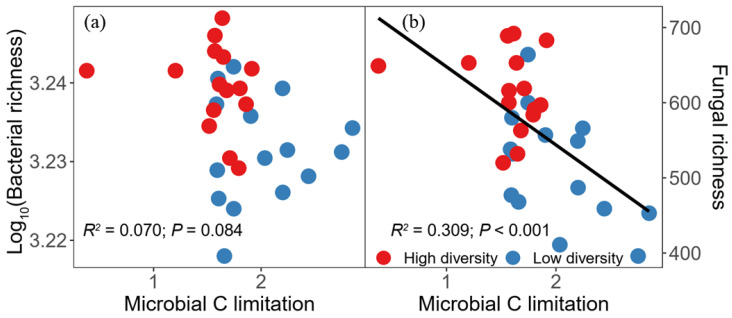
Relationships between soil microbial carbon (C) limitation and bacterial richness (**a**) and fungal richness (**b**). The black lines represent the fitted ordinary least squares (OLS) linear regressions. Red circles represent high plant diversity, while blue ones indicate low plant diversity.

**Figure 5:**
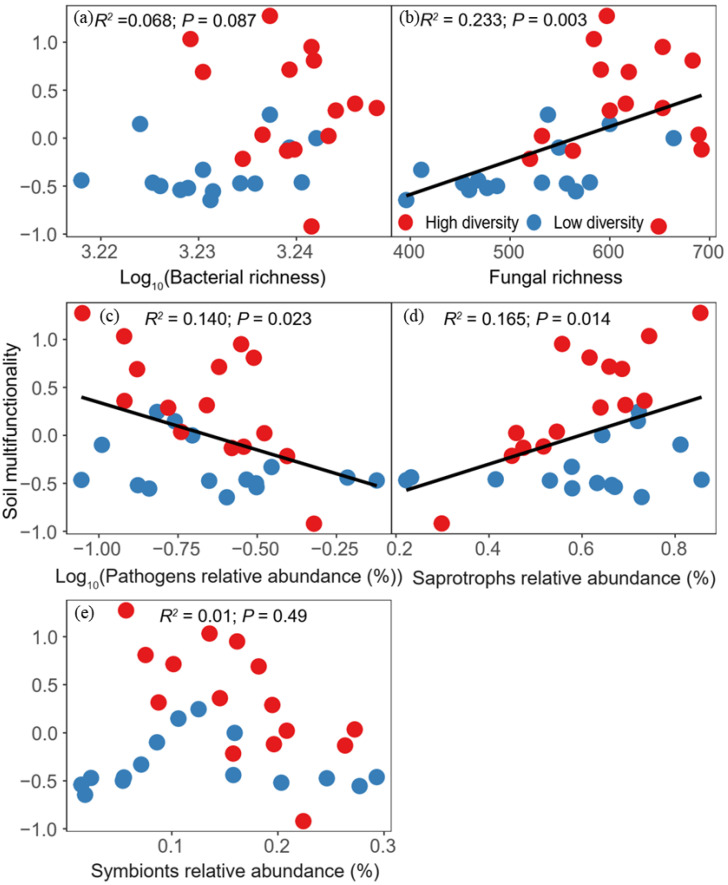
Relationships between soil multifunctionality and bacterial richness (**a**), fungal richness (**b**), pathogens relative abundance (**c**), saprotrophs relative abundance (**d**), and symbionts relative abundance (**e**). The black lines represent the fitted ordinary least squares (OLS) linear regressions. Red circles represent high plant diversity, while blue ones indicate low plant diversity.

The OLS regression models showed that microbial C limitation was significantly and negatively related to fungal richness (Figure 4b; *R*^2^ = 0.309, *p* < 0.001). Furthermore, the results showed that fungal richness was significantly and positively associated with average soil multifunctionality (Figure 5b; *R*^2^ = 0.233, *p* = 0.003). However, bacterial richness showed no correlation with soil multifunctionality (Figure 5a). Based on the positive correlation between fungal richness and soil multifunctionality, we aimed to identify the relationships between fungal guilds and soil multifunctionality. A negative linear correlation was found between the relative abundance of pathogens and soil multifunctionality (Figure 5c; *R*^2^ = 0.140, *p* = 0.023). However, we found a significantly positive relationship between the relative abundance of saprotrophs and soil multifunctionality (Figure 5d; *R*^2^ = 0.165, *p* = 0.014).

**Figure 6:**
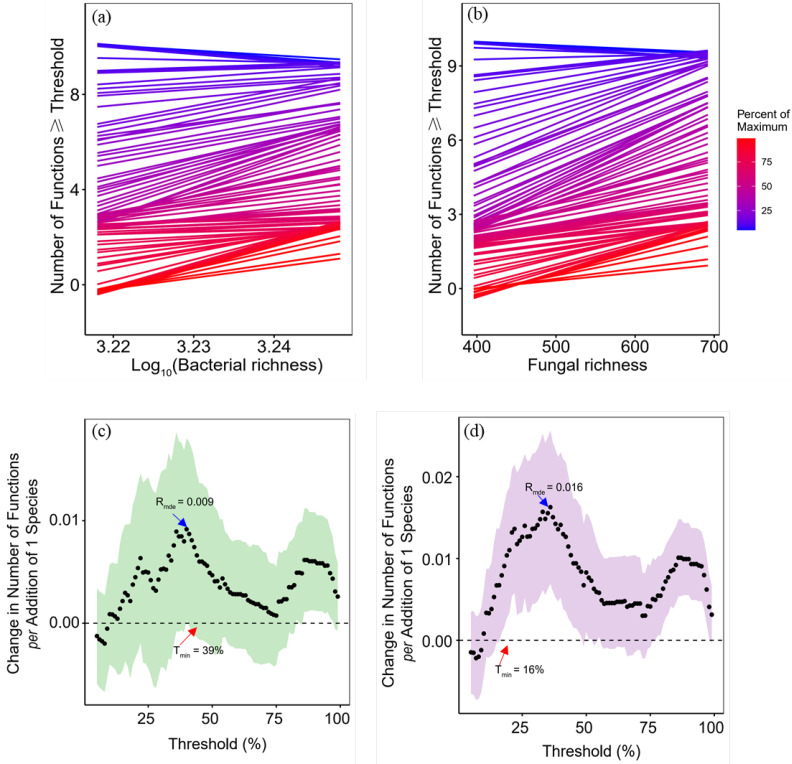
Effects of bacterial (**a**) and fungal (**b**) richness on the number of functions above thresholds. Lines represent the slope between soil microbial richness and the number of functions greater than or equal to a threshold value ranging from 5% to 99% of the maximum for each function. The dotted curves indicate the changes in the number of functions per unit increment of the richness of bacteria (**c**) and fungi (**d**). T_min_ is the minimum threshold that soil multifunctionality becomes influenced by changes in microbial richness, and R_med_ is the realized maximum effect of richness on soil multifunctionality.

Soil multifunctionality was positively and significantly related to fungal richness rather than bacterial richness. This result was confirmed using the single-threshold method (Figure S7) and the multiple-threshold method (Figure 6). Threshold methods were also used to assess whether multiple functions were performed at high levels simultaneously. Specifically, we performed single-threshold analysis; positive and significant correlations were found between the bacterial richness and soil multifunctionality at given thresholds of 75% and 90% (Figure S7g,h; *R*^2^ = 0.108, *p* = 0.042, *R*^2^ = 0.105, *p* = 0.044). However, there were significant and positive correlations between the fungal richness and soil multifunctionality at given thresholds of 25%, 50%, 75%, and 90% (Figure S7a–d; *R*^2^ = 0.220, *p* = 0.005, *R*^2^ = 0.111, *p* = 0.039, *R*^2^ = 0.313, *p* < 0.001, *R*^2^ = 0.292, *p* = 0.001). However, the multiple-threshold method does not require a threshold to be set and studies a continuous threshold gradient (Figure 6a,b). The minimum threshold (T_min_) for fungi was 16%, the lowest threshold at which fungal richness began to have a positive effect on soil multifunctionality. When the threshold was 36%, the maximum richness effect (R_mde_) was 0.016, i.e., the relationship strength of fungal richness with the strongest positive effect, indicating that the addition of one species of fungus could increase the function by 0.016 (Figure 6d).

### 3.4. Microbial Taxa Predicting Soil Multifunctionality

**Figure 7:**
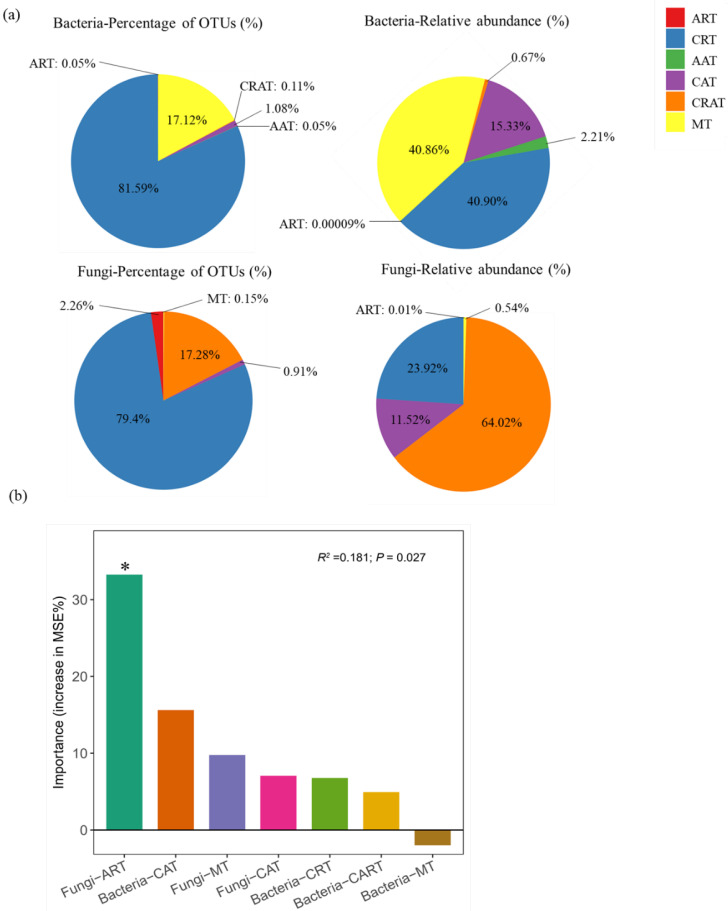
The percentage of OTUs and relative abundance of six categories of microbial taxa (**a**). The random forest regression model indicates the main microbial drivers of soil multifunctionality (**b**). MSE is the mean square error. This accuracy importance measure is computed for each tree and averaged over the forest (5000 trees) and determined by the increase in the MSE. * *p* < 0.05 on the bar shows that the associated microbial taxa have a significant effect on soil multifunctionality. ART: always rare taxa; CRT: conditionally rare taxa; AAT: always abundant taxa; CAT: conditionally abundant taxa; CRAT: conditionally rare and abundant taxa; MT: moderate taxa.

Bacterial rare taxa accounted for 81.64% of total OTUs and 40.90% of the relative abundance, respectively (Figure 7a). In comparison, abundant bacterial taxa accounted for 1.13% of total OTUs and 17.55% of the relative abundance, respectively (Figure 7a). In addition, bacterial conditionally rare and abundant taxa accounted for 0.11% of total OTUs and 0.67% of the relative abundance, respectively (Figure 7a). Bacterial moderate taxa accounted for 17.12% of total OTUs and 40.86% of the relative abundance, respectively (Figure 7a). Fungal rare taxa accounted for 81.66% of total OTUs and 23.93% of the relative abundance, respectively (Figure 7a). Fungal abundant taxa accounted for 0.91% of total OTUs and 11.52% of the relative abundance, respectively (Figure 7a). Finally, fungal conditionally rare and abundant taxa accounted for 17.28% of total OTUs and 64.02% of the relative abundance, respectively (Figure 7a). Fungal moderate taxa accounted for 0.15% of total OTUs and 0.54% of the relative abundance, respectively (Figure 7a). Random forest modeling results indicated that fungal ART (*p* = 0.012) was the most important microbial taxa in predicting soil multifunctionality (Figure 7b).

### 3.5. Direct and Indirect Effects of Plant Diversity on Soil Multifunctionality

**Figure 8:**
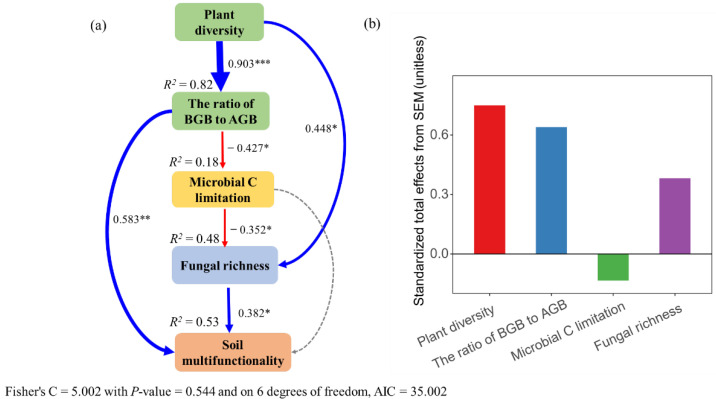
The piecewise structural equation model was used to test the direct and indirect relationships between plant diversity and soil multifunctionality (**a**). The corresponding values of the solid line arrows are the width of normalized path system arrows that reflect the size of the normalized path coefficient. The blue and red arrows show significant positive and negative correlations, respectively (* *p* < 0.05, ** *p* < 0.01, and *** *p* < 0.001). Dashed lines show non-significant relationships. The values above each variable represent the explanatory degree (*R*^2^) of each variable in the model. Standardized total effects (direct plus indirect effects) derived from the piecewise structural equation model (**b**).

The SEM analysis explained 53% of the total variation in soil multifunctionality (Figure 8a). Plant diversity indirectly affected soil multifunctionality by influencing the ratio of BGB to AGB, microbial C limitation, and fungal richness but not bacterial richness (Figure 8a and Figure S3). Plant diversity, the ratio of BGB to AGB, and fungal richness displayed positive effects on soil multifunctionality (Figure 8b). However, microbial C limitation exhibited a negative effect on soil multifunctionality (Figure 8b).

## 4. Discussion

Our study provides evidence that fungal richness rather than bacterial richness was significantly related to soil multifunctionality in a semi-arid grassland in northeast China. Saprotrophic fungi and rare fungal taxa were essential for maintaining soil functions. Furthermore, under high diversity plant assemblages, the changes in plant biomass allocation patterns increased plant below-ground biomass, which can alleviate microbial C limitation and thus enhance the fungal richness, ultimately promoting soil multifunctionality. Such results suggest that the above-ground and below-ground biodiversity, as well as rare fungal taxa, are vital to maintaining ecosystem functions in a semi-arid grassland ecosystem.

Soil microorganisms are some of the most sensitive components to precipitation change in semi-arid grasslands [69]. Therefore, biodiversity in semi-arid zones can be seriously compromised if rainfall changes. For example, reducing precipitation significantly increased fungal diversity [70]. However, one study showed a significant positive correlation between fungal diversity and precipitation [71]. Previous studies have suggested that bacterial communities are more sensitive to changes in precipitation than fungal communities in a semi-arid grassland [72]. However, we found that fungal richness and saprotrophic fungi were principal biotic factors in regulating soil multifunctionality in a semi-arid grassland. Fungi can create an environment around themselves by secreting polysaccharides from their hyphae to prevent dehydration [73]. Moreover, substrate diffusion restrictions might force the soil fungal mycelium network to expand, which is conducive to absorbing water and nutrients [74]. Therefore, fungi are more resistant to drought than bacteria, which may play an important role in arid and semi-arid grasslands [75]. For instance, soil fungi are crucial to organic matter decomposition, and root-associated fungi are important regulators of ecosystem C dynamics [22]. Moreover, fungal richness was significantly positively correlated with denitrification activity, indicating that fungi could promote soil N cycling [14]. Our results also showed that saprotrophic fungi significantly affected soil multifunctionality (Figure 5d). Saprotrophic fungi mainly grow throughout the soil litter interface and are involved in plant litter decomposition [76]. Previous studies have also suggested that saprotrophic fungi might affect soil C storage through interactions with ectomycorrhizal fungi [77]. Moreover, free-living saprotrophs generally play an essential ecological role in dead plant material because they can derive C by propagules or hyphae from dead organic material [78]. Saprotrophic fungi can also obtain fresh nutrients from recalcitrant organic polymers using extracellular enzymes and the nonenzymatic Fenton reaction [79,80]. For example, *Podospora anserina* is a potent plant biomass degrader and efficiently utilizes lignocellulose as a C source through dedicated lignin degradation enzymes [81,82]. Therefore, the ability of saprophytic fungi to translocate C resources implies that saprophytic fungi are essential agents of nutrient redistribution in soils [83]. Furthermore, the hyphal network formed by saprotrophic fungi is involved in the formation of soil aggregates, which is of great significance for soil water retention and erosion resistance [84]. Collectively, the present study suggested the non-negligible roles played by the fungal richness and saprophytic fungi in regulating soil functions.

An interesting result was that fungal ART was identified as the main driver of soil multifunctionality (Figure 7b). Previous studies have suggested that rare fungal community composition and functional guilds are more stable than those of the abundant taxa under certain conditions [85]. In other words, rare taxa contribute to maintaining the microbiome’s function under environmental stress because some may be highly resistant to stress [86]. For example, a recent study found that rare microbial taxa might modulate the adverse effects of semi-arid grassland degradation drivers such as vegetation loss and eutrophication on soil organic matter decomposition [24]. Furthermore, rare taxa contributed more to soil C and N cycling and crop yield than the abundant taxa [25]. Thus, rare microbial taxa play a unique role in maintaining ecosystem functions [28,87]. However, our understanding of rare microbial taxa is still preliminary, and more attention should be given to rare microbial taxa in future studies of biodiversity and ecosystem multifunctionality.

**Figure 9:**
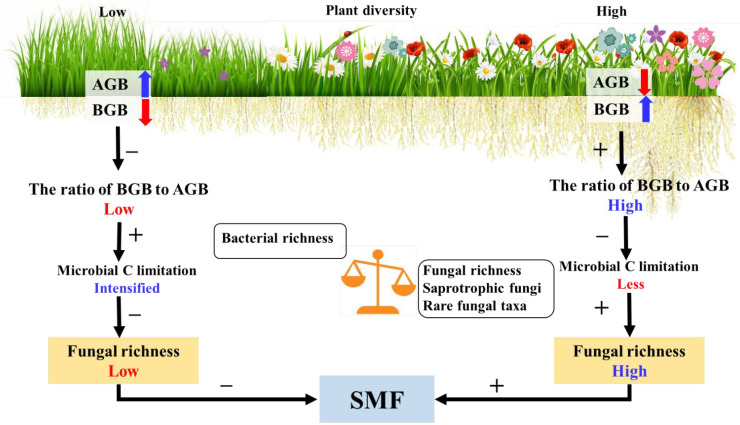
A conceptual framework for understanding the effects of plant and fungal richness on soil multifunctionality in a semi-arid grassland. Changing plant biomass allocation patterns increased the ratio of plant below-ground biomass to above-ground biomass under high diversity plant assemblages, which can alleviate microbial carbon (C) limitation and thus enhance the fungal richness, finally promoting soil multifunctionality. The fungal richness was positively related to soil multifunctionality, but the bacterial richness was not. Saprotrophic fungi and rare fungal taxa were essential for maintaining the soil functions. The blue arrow represents an increase, and the red arrow represents a decrease. + and − describe promotion and inhibition effects. AGB: plant above-ground biomass; BGB: plant below-ground biomass; SMF: soil multifunctionality.

As expected, in line with previous studies, high plant diversity increased soil multifunctionality as compared with low plant diversity [4,56,88]. However, our study elucidated the underlying mechanisms by which high plant diversity could enhance soil multifunctionality through fungal richness. According to current knowledge [89], the possible reason was that the competition for below-ground resources was less than that for above-ground resources in low diversity plant assemblages. Therefore, low diversity plant assemblages might increase the allocation of above-ground biomass to compete for light [89]. Although above-ground biomass was expected to be associated with litter production, below-ground C from rhizodeposition was vital for soil microbial communities [90]. C decomposes from above-ground plant litter to the soil surface; thus, it is not readily available to microorganisms inside the soil [36]. Therefore, a low diversity plant assemblage might intensify microbial C limitation [91], mainly by changes in plant-root-derived substrates [37]. Our results indicated that microbial C limitation significantly affected soil fungal richness but not bacterial richness (Figure 4a,b). A reasonable explanation might be that members of the fungal community are the first consumers of below-ground plant-derived C inputs to the soil [24,92]. Thus, intensifying microbial C limitation reduced soil fungal richness and ultimately decreased soil multifunctionality in the low diversity plant assemblage (Figure 9). However, high diversity plant assemblages increased below-ground biomass allocation (Figure 2a). A possible explanation for the increase in below-ground biomass may be that the interactions between different plant species roots may affect biomass allocation patterns, i.e., interspecific competition can increase plant below-ground biomass [93]. The total soil organic carbon content under high plant diversity was higher than that under low plant diversity in this study area (Figure S3g). Although this study did not test the contents of soil organic matter mineralization, previous literature has shown that the quantity and quality of soil organic matter are the main driving factors affecting microorganisms [94]. The increasing plant below-ground biomass may improve the quantity and quality of soil organic matter in semi-arid zones [32], and the increased soil C resources provide rich substrates and energy for microorganism growth and basal metabolism [7], thus increasing fungal diversity. In this study, the decreased microbial C limitation favored fungal richness under high plant diversity, thus promoting soil multifunctionality (Figure 9). Furthermore, our results indicate that semi-arid grassland provides essential ecosystem services such as carbon sequestration, climate regulation, and biodiversity protection under high diversity plant assemblages. Our findings suggest that high diversity plant assemblages make semi-arid grassland ecosystems more efficient in regulating ecological processes, which local stakeholders or grassland conservation agencies want. Therefore, ecosystem services through the TESSA methodology (http://tessa.tools/) (accessed on 1 June 2021) [95] should contain future multifunctionality studies. The study of ecosystem services multifunctionality can provide important enlightenment for ecological protection and sustainable development under the increasing pressure of human activities and climate changes. In summary, this study revealed the mechanism of changes in soil multifunctionality in which plant-diversity-induced alterations of microbial C limitation regulate soil multifunctionality by affecting soil fungal richness in a semi-arid grassland.

## 5. Conclusions

Our results demonstrated that the positive effect of plant diversity on soil multifunctionality was mainly due to the high plant diversity increasing plant below-ground biomass allocation, which alleviated microbial C limitation and favored fungal richness, finally promoting soil multifunctionality. Additionality, the high diversity of plant assemblages enhanced ecosystem services for semi-arid grasslands protection, and plant-fungus relationships were important to improve the assessment of ecosystem services. Instead of bacterial richness, the fungal richness was the crucial biotic predictor of soil multifunctionality in a semi-arid grassland. Furthermore, saprotrophic fungi and rare fungal taxa were major drivers of soil multifunctionality. In conclusion, this study provides a new perspective for evaluating the relative roles of fungal and bacterial diversity and biomass allocation patterns in maintaining soil functions in the context of global plant diversity loss and has important implications for biodiversity conservation and sustainable development in semi-arid grasslands. Ecosystem multifunctionality and ecosystem services multifunctionality should be popularized and applied in future multifunctionality studies. 

## Data Availability

The data supporting this study’s findings are available from the corresponding author upon reasonable request.

## Supplementary Materials

The following supporting information can be downloaded at: https://www.mdpi.com/article/10.3390/biology11060870/s1. Table S1: The plant species composition of the selected low diversity with a single species and high diversity with multiple species assemblages in the Songnen grassland, China; Table S2: Variables used to estimate soil multifunctionality and their importance; Figure S1: Principal component analysis (PCA) to determine the difference of soil characteristics between low diversity plant assemblages and high diversity plant assemblages. The soil characteristics included soil water content, soil pH, soil electrical conductivity, soil available nitrogen, total soil nitrogen, total soil phosphorus, soil total organic carbon, and soil C/N; Figure S2: *Prior* structural equation models (SEM) of hypothetical relationships between plant diversity and soil multifunctionality (a). Models were accepted for the significant Fisher’s C test (*p* > 0.05). Among significant models, the one with the lowest Akaike information criterion (AIC) was selected for the final SEM analysis (b–d); Figure S3: Soil water content (SWC) (a), soil pH (b), soil electrical conductivity (EC) (c), soil available nitrogen (AN) (d), soil total nitrogen (TN) (e), soil total phosphorus (TP) (f), soil total organic carbon (TOC) (g), and soil C/N (h) in response to low and high plant diversities. * *p* < 0.05, ** *p* < 0.01, and *** *p* < 0.001 (*t*-test); Figure S4: Rarefaction curves for the soil bacterial (a) and fungal (b) communities at 97% sequence similarity in different plant diversity assemblages. Low diversity assemblages including single species *Carex duriuscula* C.A.Mey. (CD), *Lespedeza hedysaroides* (Pall.) Kitag. (LH), and *Calamagrostis rigidula* A.I.Baranov and Skvortsov (CR) assemblages. High diversity assemblages including multiple species assemblages with the dominant *Lespedeza daurica* (Laxm.) Schindl. (LD.ass), *Leymus chinensis* (Trin.) Tzvelev (LC.ass), and *Hierochloe glabra* Trin. (HG.ass); Figure S5: The relative abundance of bacterial and fungal communities at the phylum level. “Others” represents all phyla with relative abundance < 1%. ** *p* < 0.01, and *** *p* < 0.001 (*t*-test); Figure S6: Fungal (a) and bacterial richness (b) in response to plant diversity. *** *p* < 0.001 (*t*-test); Figure S7: The relationship between fungal (a–d) and bacterial richness (e–h) and soil multifunctionality, at four different thresholds 25%, 50%, 75%, and 90% of maximum. The black lines represent the fitted ordinary least squares (OLS) linear regressions. Red circles represent high plant diversity, while blue ones indicate low plant diversity.
