# Supplementary material for: Plant Diversity and Fungal Richness Regulate the Changes in Soil Multifunctionality in a Semi-Arid Grassland
Source: Biology (Basel). 2022 Jun 6;11(6):870. doi: 10.3390/biology11060870 (PMC9220314; doi:10.3390/biology11060870)
Supplement: Supplementary file 1 [file biology-11-00870-s001.zip › biology-1738780-supplementary.pdf]

Table S1. The plant species composition of the selected low diversity with a single species and high diversity with multiple species assemblages in the Songnen grassland, China.

Plant species	Low diversity with single species assemblages			High diversity with multiple species assemblages		
	Carex duriuscula C.A.Mey.	*Lespedeza hedysaroides* (Pall.) Kitag.	*Calamagrostis rigidula* A.I.Baranov & Skvortsov	*Lespedeza daurica* (Laxm.) Schindl.	*Leymus chinensis* (Trin.) Tzvelev	*Hierochloe glabra* Trin.
Leymus chinensis	-	-	-	+	+	+
Lespedeza daurica	-	-	-	+	+	+
Lespedeza hedysaroides	-	+	-	-	+	+
Cynanchum thesioides K.Schum.	-	-	-	+	+	-
Clalamagrostis rigidula	-	-	+	-	+	-
Potentilla flagellaris D.F.K.Schltdl.	-	-	-	+	+	+
Hierochloe glabra	-	-	-	-	+	+
Carex duriuscula	+	-	-	+	+	+
Artemisia scoparia Waldst. & Kit.	-	-	-	+	+	-
Calamagrostis macrolepis Litv.	-	-	-	+	-	-
Astragalus adsurgens Willd. ex Steud.	-	-	-	+	-	-
Cleistogenes squarrosa (Trin.) Keng	-	-	-	+	-	-
Medicago ruthenica Trautv.	-	-	-	+	-	-
Ixeris polycephala Cass.	-	-	-	+	-	-
Convolvulus sagittifolius Salisb.	-	-	-	+	-	-
Astragalus scaberrimus Bunge	-	-	-	+	-	-
Setaria viridis (L.) P.Beauv.	-	-	-	+	-	-
Chenopodium album Bosc. ex Moq.	-	-	-	+	-	-
Artemisia carvifolia Besser	-	-	-	-	-	+

+ and - indicate the presence and absence of this plant in the plant assemblages.

Table S2. Variables used to estimate soil multifunctionality and their importance.

Variables	Importance
Carbon cycling	
α-1,4-glucosidase	
β-1,4-glucosidase	Climate regulation and play roles in the global carbon
β-1,4-xylosidase	cycle
β-D-cellobiohydrolase	
Nitrogen cycling	
Leucine aminopeptidase	
β-1,4-N-acetylglucosaminidase	Climate regulation and play roles in the global nitrogen
Net nitrification rate	cycle
Net N mineralization rate	
Phosphorous cycling	
Alkaline phosphatase	Climate regulation and play roles in the global
Available phosphorus	phosphorous cycle

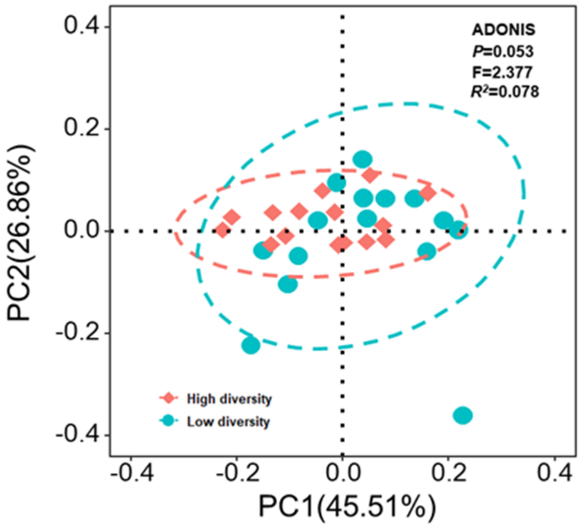

Figure S1. Principal component analysis (PCA) to determine the difference of soil characteristics between low diversity plant assemblages and high diversity plant assemblages. The soil characteristics included soil water content, soil pH, soil electrical conductivity, soil available nitrogen, soil total nitrogen, soil total phosphorus, soil total organic carbon, and soil C/N.

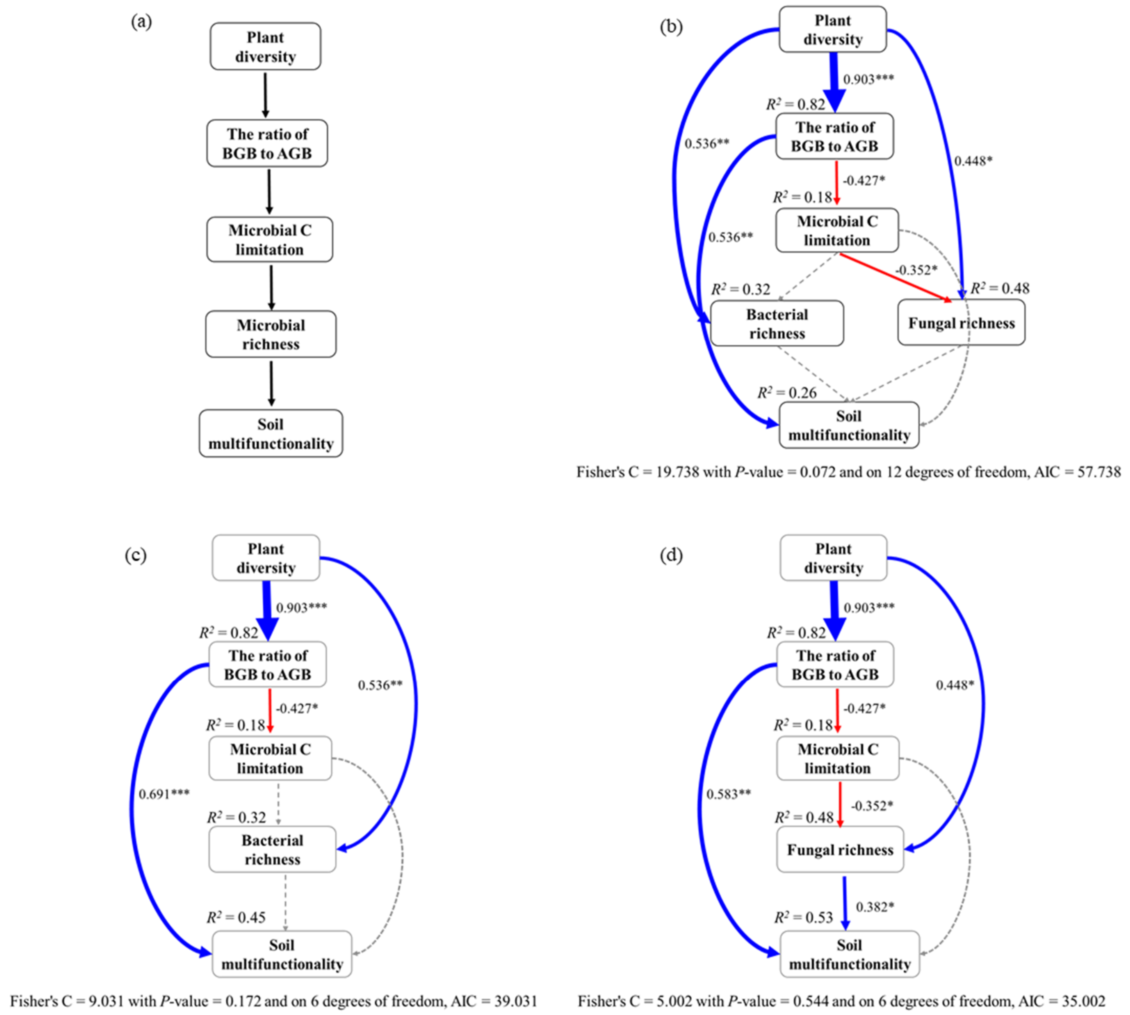

Figure S2. *Prior* structural equation models (SEM) of hypothetical relationships between plant diversity and soil multifunctionality (a). Models were accepted for significant Fisher's C test ($P > 0.05$). Among significant models, the one with the lowest Akaike information criterion (AIC) was selected for the final SEM analysis (b-d).

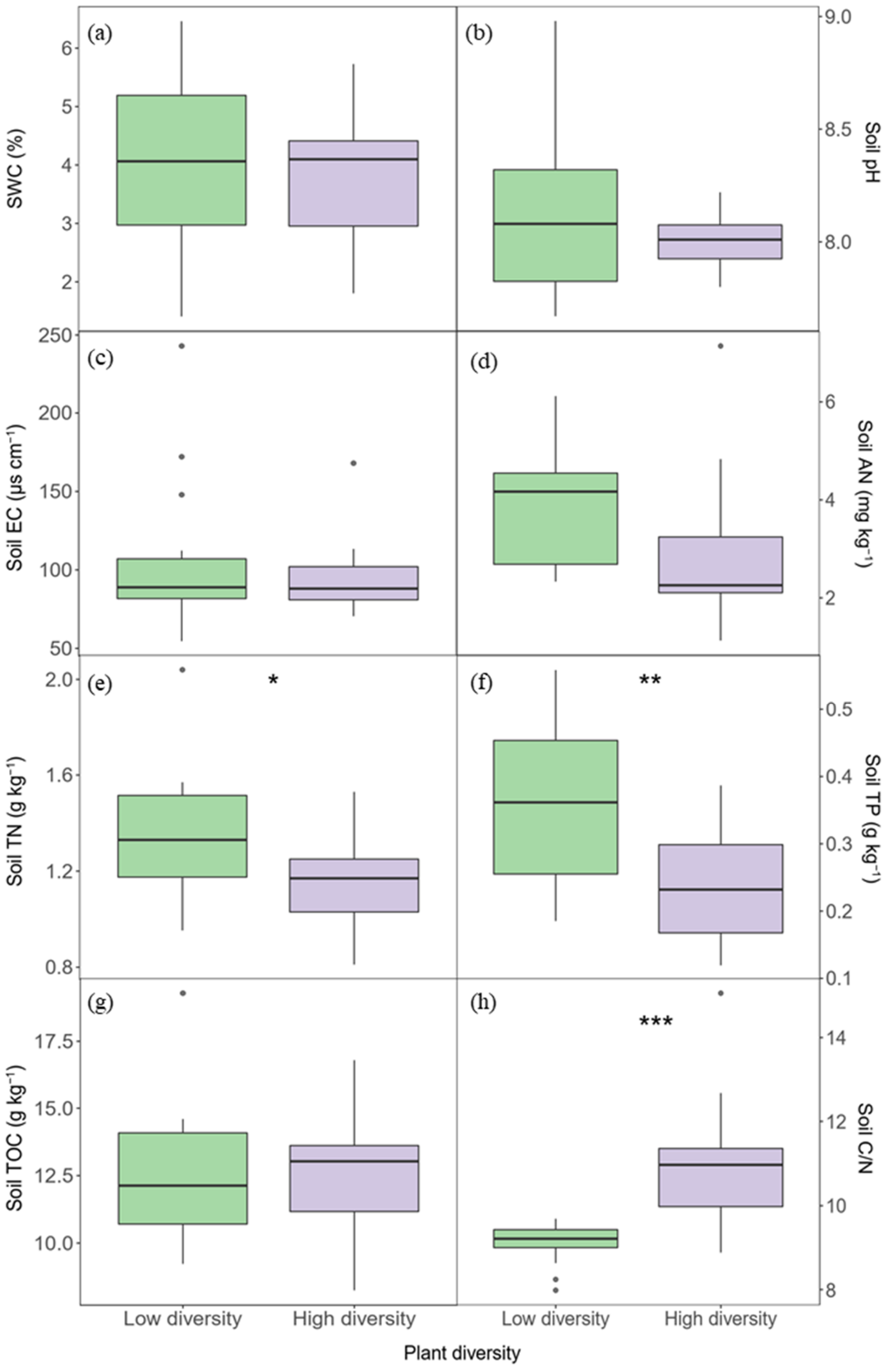

Figure S3. Soil water content (SWC) (a), soil pH (b), soil electrical conductivity (EC) (c), soil available nitrogen (AN) (d), soil total nitrogen (TN) (e), soil total phosphorus (TP) (f), soil total organic carbon (TOC) (g), and soil C/N (h) in response to low and high plant diversities. $^{*}P$ < 0.05, $^{**}P$ < 0.01, and $^{***}P$ < 0.001 (*t*-test).

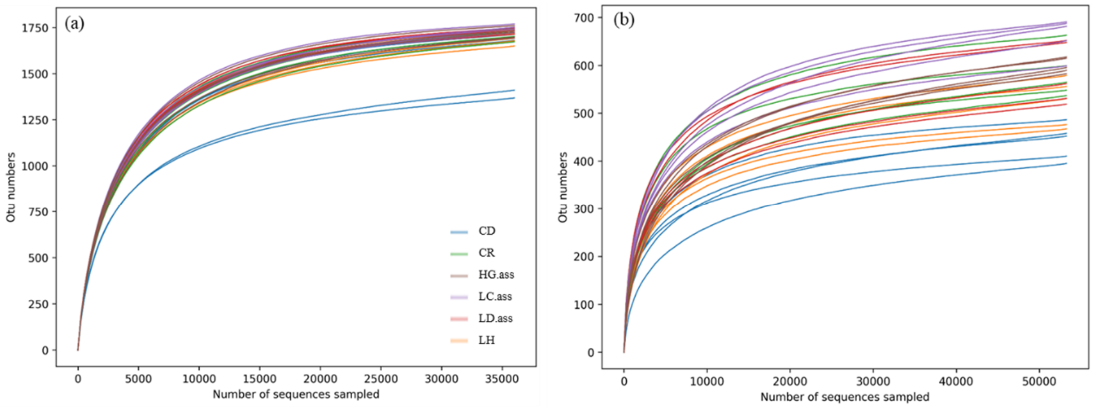

Figure S4. Rarefaction curves for the soil bacterial (a) and fungal (b) communities at 97% sequence similarity in different plant diversity assemblages. Low diversity assemblages including single species *Carex duriuscula* C.A.Mey. (CD), *Lespedeza hedysaroides* (Pall.) Kitag. (LH), and *Calamagrostis rigidula* A.I.Baranov & Skvortsov (CR) assemblages. High diversity assemblages including multiple species assemblages with the dominant *Lespedeza daurica* (Laxm.) Schindl. (LD.ass), *Leymus chinensis* (Trin.) Tzvelev (LC.ass), and *Hierochloe glabra* Trin. (HG.ass).

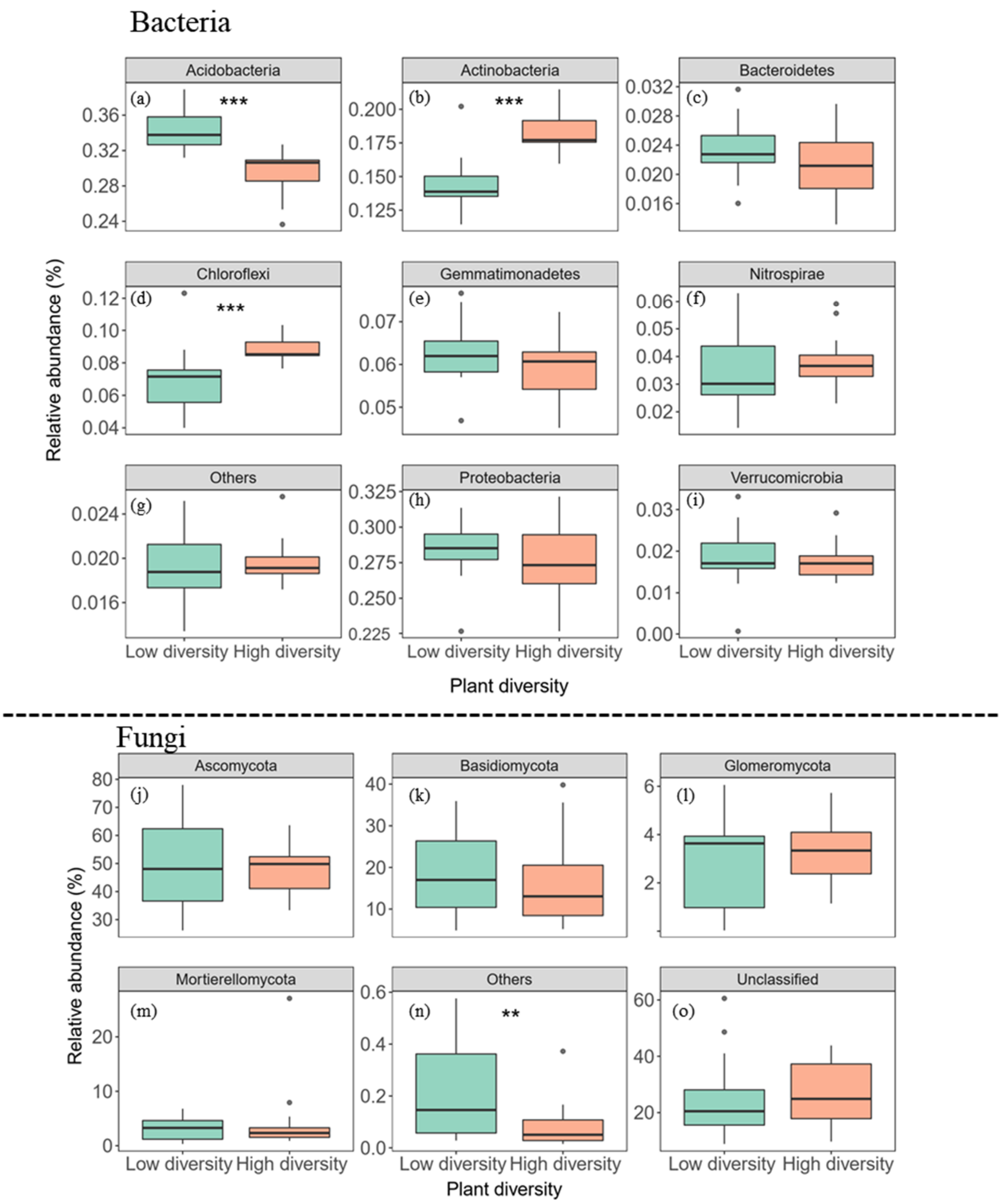

Figure S5. The relative abundance of bacterial and fungal communities at phylum level. “Others” represents all phyla with relative abundance < 1%. $^{**}P < 0.01$, and $^{***}P < 0.001$ (*t*-test).

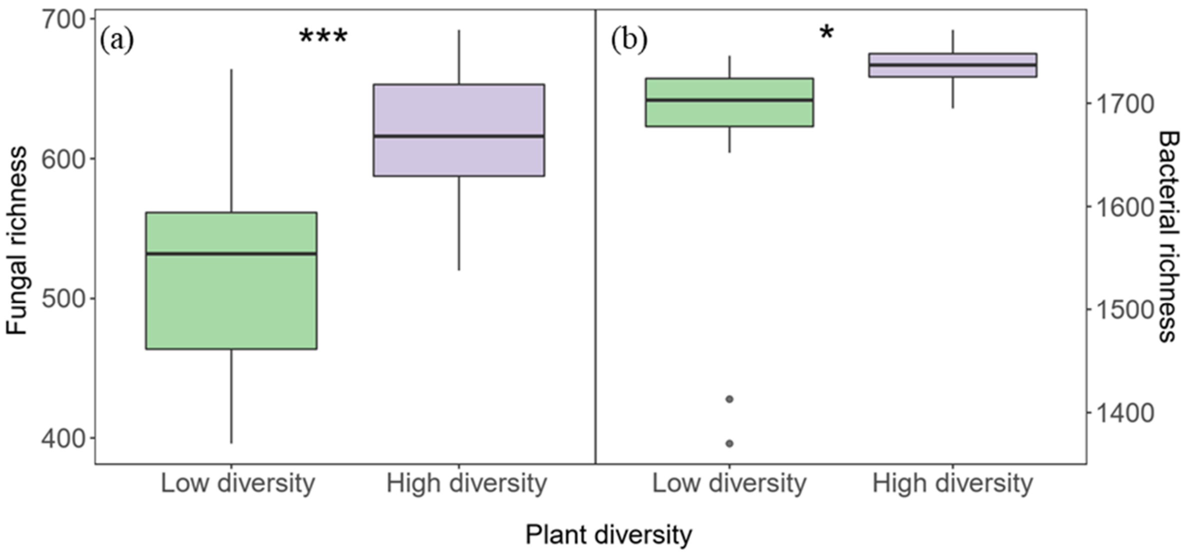

Figure S6. Fungal and bacterial richness in response to plant diversity. ***$P < 0.001$ (t-test).

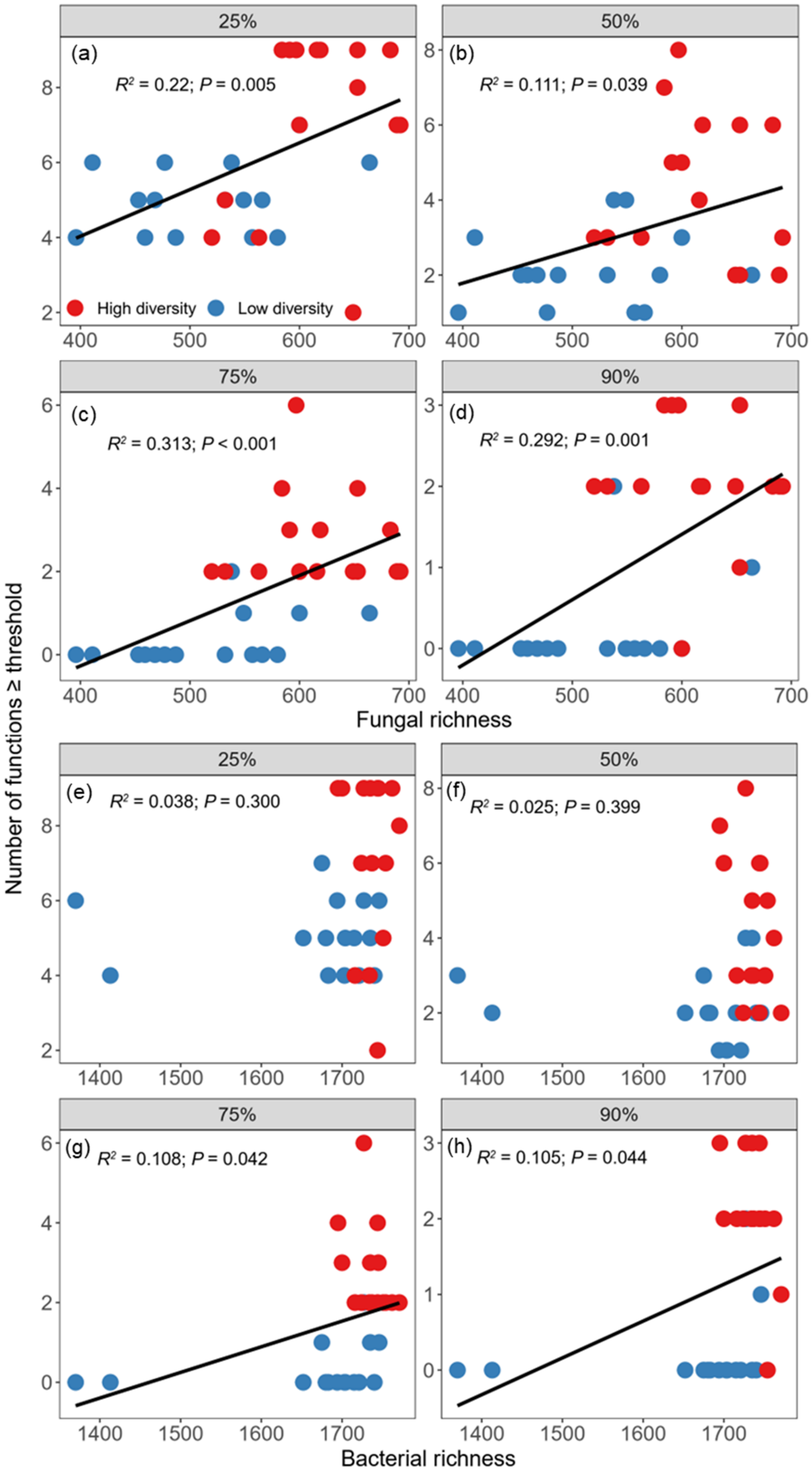

Figure S7. The relationship between fungal (a-d) and bacterial richness (e-h) and soil multifunctionality, at four different thresholds 25%, 50%, 75%, and 90% of maximum. The black lines represent the fitted ordinary least squares (OLS) linear regressions. Red circles represent high plant diversity while blue ones indicate low plant diversity.
